# BMI-related cortical morphometry changes are associated with altered white matter structure

**DOI:** 10.1038/s41366-018-0269-9

**Published:** 2018-12-19

**Authors:** Nenad Medic, Peter Kochunov, Hisham Ziauddeen, Karen D. Ersche, Pradeep J. Nathan, Lisa Ronan, Paul C. Fletcher

**Affiliations:** 1 Department of Psychiatry, University of Cambridge, Cambridge, CB2 8AH UK; 2 Wellcome Trust-MRC Institute of Metabolic Science, University of Cambridge, Cambridge, CB2 0QQ UK; 3 Maryland Psychiatric Research Center, University of Maryland School of Medicine, Baltimore, MD USA; 4 Cambridgeshire & Peterborough NHS Foundation Trust, Cambridge, CB21 5EF UK; 5 School of Psychological Sciences, Monash University, Melbourne, Australia; 6 Heptares Therapeutics Ltd, Cambridge, UK

**Keywords:** Cognitive neuroscience, Anatomy

## Abstract

**Background:**

While gross measures of brain structure have shown alterations with increasing body mass index (BMI), the extent and nature of such changes has varied substantially across studies. Here, we sought to determine whether small-scale morphometric measures might prove more sensitive and reliable than larger scale measures and whether they might offer a valuable opportunity to link cortical changes to underlying white matter changes. To examine this, we explored the association of BMI with millimetre-scale Gaussian curvature, in addition to standard measures of morphometry such as cortical thickness, surface area and mean curvature. We also assessed the volume and integrity of the white matter, using white matter signal intensity and fractional anisotropy (FA). We hypothesised that BMI would be linked to small-scale changes in Gaussian curvature and that this phenomenon would be mediated by changes in the integrity of the underlying white matter.

**Methods:**

The association of global measures of T1-weighted cortical morphometry with BMI was examined using linear regression and mediation analyses in two independent groups of healthy young to middle aged human subjects (*n*_1_ = 52, *n*_2_ = 202). In a third dataset of (*n*_3_ = 897), which included diffusion tensor images, we sought to replicate the significant associations established in the first two datasets, and examine the potential mechanistic link between BMI-associated cortical changes and global FA.

**Results:**

Gaussian curvature of the white matter surface showed a significant, positive association with BMI across all three independent datasets. This effect was mediated by a negative association between the integrity of the white matter and BMI.

**Conclusions:**

Increasing BMI is associated with changes in white matter microstructure in young to middle-aged healthy adults. Our results are consistent with a model whereby BMI-linked cortical changes are mediated by the effects of BMI on white matter microstructure.

## Introduction

Obesity is recognised as a risk factor for dementia in the elderly population [1] and impaired cognitive function in children and adults [2]. As a consequence, there has been an increasing focus on understanding the links between body mass index (BMI) and changes in brain structure. With the focus predominantly on the brain’s gray matter, the majority of studies have found evidence of alterations but the extent and nature of the changes has varied substantially across studies [3].

The same is true of white matter changes. For example, obesity has been associated with greater white matter volumes in frontal, temporal and parietal lobes [4], as well as in the limbic system, brain stem and cerebellum [5]. Conversely, other studies have reported a negative relationship between BMI and white matter volumes of basal ganglia and corona radiata [6, 7]. This inconsistency may be due to the non-linear trajectory of age-related changes in white matter volume, which may obscure the obesity-related differences. Indeed, we have recently demonstrated that obesity is associated with an increase in age-related white matter loss [8].

To clarify these findings, the microstructure of white matter has been investigated using diffusion tensor imaging (DTI), a sensitive tool for assessing the integrity of white matter based on mapping the directionality of water movement within white matter fibres [9]. The exploration of fractional anisotropy (FA), the most widely used DTI marker of white matter integrity, has yielded more consistent evidence linking obesity to differences in white matter structure. Indeed, a negative association between BMI and FA has been repeatedly demonstrated in tracts within the limbic system and those connecting the temporal and frontal lobes (reviewed in Kullman et al [10]).

Here, we sought to make use of convergent and complementary measures of gray and white matter morphometry to more clearly understand the underlying mechanisms through which global brain changes may occur in the context of obesity. It has previously been reported that subtle differences in cortical features in subjects with mild cognitive impairment and Alzheimer’s disease are driven by reduced white matter volume and white matter integrity [9]. Based on this report and geometric considerations, we hypothesised that altered white matter microstructure might be the mediating factor between obesity and changes in the cerebral cortex.

**Fig. 1:**
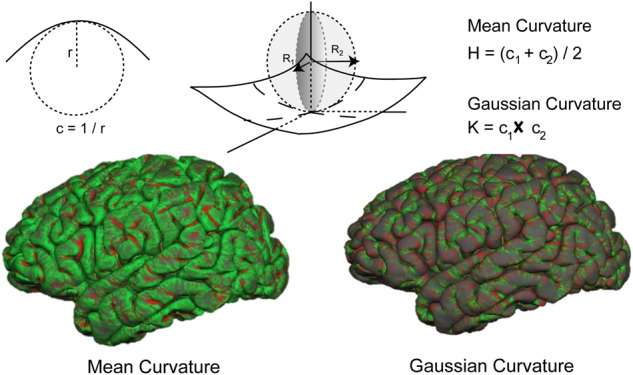
The curvature, c, at a point on a line is defined as the inverse of the radius of the osculating circle at that point. On a surface, the curvature of each point is a function of principal curvatures at that point, which are always orthogonal to each other. The mean curvature is the average of the principal curvatures, while the Gaussian curvature is the product of principal curvatures. Presented here are the maps of mean and Gaussian curvatures of the cortical surface reconstruction. While the mean curvature follows the pattern of gyri (in green) and sulci (in red), the pattern of Gaussian curvature is of much higher spatial frequency and does not follow the larger-scale morphological features of cortical folds. Positive Gaussian curvature is depicted in red and negative Gaussian curvature in green. Taken from [12]

We tested this hypothesis using measurements of gray and white matter morphometry. Specifically, we predicted that BMI-associated changes in white matter microstructure mediated the cortical changes detectable as the increase in the degree of Gaussian curvature at the brain’s surface. While the so-called mean or extrinsic curvature of a surface depicts its extrinsic shape, i.e., the folding of the cortex, Gaussian curvature is an intrinsic feature of the surface (Fig. 1) and may be used to quantify the stretching or deformation of a surface. Gaussian curvature is measured at the millimeter scale and has been shown to be highly sensitive to morphometric differences [11, 12]. For example, it has previously been used to identify subtle differences in cortical features of patients affected by schizophrenia compared to healthy controls [13], changes related to autism [14], and in a study of healthy participants with different polymorphisms of the brain-derived neurotrophic factor (BDNF) [15].

Based on previously published work, we hypothesised that obesity is primarily associated with subtle changes in the microstructure of white matter, and therefore that the measure of millimeter-scale Gaussian curvature would be a more sensitive indirect marker of subtle BMI-driven changes in white matter microstructure than mean curvature. We studied the relationship between BMI and cortical morphometric measures in three independent datasets (A, B and C) of healthy young to middle-aged subjects. In datasets A and B, we examined associations between BMI and T1-weighted cortical morphometry measures, including Gaussian curvature, mean curvature, surface area and cortical thickness. We extended this to assessing white matter measures: volume and signal intensity, adopted as a T1-weighted marker of white matter integrity. In dataset C, we sought to replicate significant effects established in datasets A and B, and extended our analysis by measuring FA, as a well-validated measure of white matter integrity based on DTI. Finally, across all three datasets we also conducted post-hoc mediation analyses, based on the results of the regression analyses. These were conducted to formally establish a link between BMI-linked changes in cortical and white matter measures.

## Methods

### Subjects

#### Dataset A

52 subjects (mean ± SD: age 25.44 ± 5.27 years (range 18.19–42.08 years), BMI 27.46 ± 6.11 kg/m^2^ (range 19.19–38.89 kg/m^2^), 26 females) were scanned as part of studies conducted at the Child Psychiatry Branch of the National Institute of Mental Health, in Bethesda, Maryland, US. Scanning was approved by the institutional review board of the National Institute of Mental Health. All subjects provided written informed consent. Subjects had no history of brain injury or neurological disorder. T1-weighted scans were acquired on a 1.5 T GE Sigma scanner, using a 3D SPGR sequence with the following parameters: echo time = 5 ms, repetition time = 24 ms, flip angle = 45 degrees, slice thickness = 1.5 mm, in-plane resolution of 0.9375 × 0.9375 mm, field of view 240 mm.

#### Dataset B

202 subjects (mean ± SD: age 32.29 ± 7.72 years (range 18 – 50 years), BMI 28.45 ± 6.21 kg/m^2^ (range 18.5–46.4 kg/m^2^), 158 females) were scanned as part of studies conducted at the Department of Psychiatry, University of Cambridge, Cambridge, United Kingdom. Scanning was approved by the National Health Service Local Research and University of Cambridge Psychology Research Committees. All subjects provided informed consent. Subjects self-reported as healthy, with no relevant medical history. T1-weighted scans were acquired on two 3 T scanners, the Siemens Trio and Siemens Verio, using an MP-RAGE sequence with the following parameters: echo time = 2.98 ms, repetition time = 2300 ms, inversion time = 900 ms, flip angle = 9 degrees, isotropic resolution of 1 mm, field of view 256 mm.

#### Dataset C

Structural scans from 897 subjects (mean ± SD: age 28.82 ± 3.68 years (range 22–37 years), BMI 26.65 ± 5.29 kg/m^2^ (range 16.48–47.76 kg/m^2^), 503 females) were obtained from the 2015 public release of the Human Connectome Project (HCP) (www.humanconnectome.org). This group of subjects comprised 107 monozygotic twin pairs, 116 dizygotic twin pairs and 451 fraternal siblings, from 381 families. The subjects were scanned in Washington University, St Louis, Missouri, US. A detailed list of inclusion/exclusion criteria can be found in [16]. Scanning was approved by the Washington University institutional board, and all participants provided written informed consent. Details on the scanning and processing are available online: https://www.humanconnectome.org/storage/app/media/documentation/s900/HCP_S900_Release_Reference_Manual.pdf). Briefly, T1-weighted scans were acquired on a Siemens Skyra 3 T scanner, using the 3D MPRAGE sequence, with the following parameters: echo time = 2.14 ms, repetition time = 2400 ms, flip angle = 8 degrees, isotropic resolution of 0.7 mm, field of view = 224 mm.

DTI scans were acquired on a Siemens Connectome 3 T (100 mT/m maximum gradient strength and a 32 channel head coil), using a single-shot, single refocusing spin-echo, echo-planar sequence with 0.25 mm isotropic spatial resolution (TE = 89.5 ms, TR = 5520 ms, FOV = 210 × 180 mm). Three gradient tables of 90 diffusion-weighted directions and six non-diffusion weighted images each (b = 0) were collected with right-to-left and left-to-right phase encoding polarities for each of the three diffusion weightings (*b* *=* 1000, 2000, and 3000 s/mm^2^).

### MRI processing and morphometric measures

Cortical reconstructions of T1-weighted scans (datasets A, B and C) were made in FreeSurfer. Measures of global average cortical thickness, total surface area (of white matter and pial surfaces), total white matter volume and white matter intensity at 1 mm distance along surface normal towards white matter [17] were computed in FreeSurfer. Global average mean and Gaussian curvature were calculated in the software Caret (v5.65, http://brainmap.wustl.edu/caret) and Matlab [18]. DTI data were pre-processed in the HCP Diffusion Pipeline [19]. The ENIGMA-DTI protocol, described in [20] and available online (http://enigma.ini.usc.edu/ongoing/dti-working-group/), was used to extract the whole-brain average FA values. A detailed description of MRI processing and extraction of morphometric measures is available in the Supplementary information.

#### T1-weighted white matter signal intensity and FA cross-validation (dataset C)

In order to cross-validate the measure of T1-weighted white matter signal intensity to FA, as a well-established measure of white matter integrity, we examined the correlation between these two measures in dataset C.

### Statistical analyses

All morphometric measures were converted to z-scores. In datasets A and B, linear models were used to explore the association between BMI and global measures of average thickness, total surface area (of the pial and white matter surface), total white matter volume, average white matter signal intensity, and 4 measures of curvature: global average mean and Gaussian curvature, both of the white matter and pial surfaces. To adjust for nine tests conducted, a significance threshold α = 0.05 was corrected using the false discovery rate (FDR) method. In dataset C, we re-examined the association between BMI and Gaussian curvature of the white matter surface and white matter signal intensity, and explored the association between BMI and global average FA. The significance threshold was FDR-corrected for the three comparisons made. Given that this dataset comprised groups of twins and fraternal siblings, a linear mixed effects model was used (nlme package in R), treating family membership as a random effect.

In all models, age and sex were used as covariates. In models exploring the curvature measures, surface area of the pial/white matter surfaces was also used as a covariate; in models exploring the white matter volume, intracranial volume was used as a covariate. Given that subjects’ scans in dataset B were acquired on two different scanners, type of scanner was additionally included as a covariate in all models in dataset B. Mediation analysis was conducted in the R package mediate, using 1000 simulations.

## Results

### Datasets A and B

#### Regression analyses

##### Gray matter

**Fig. 2:**
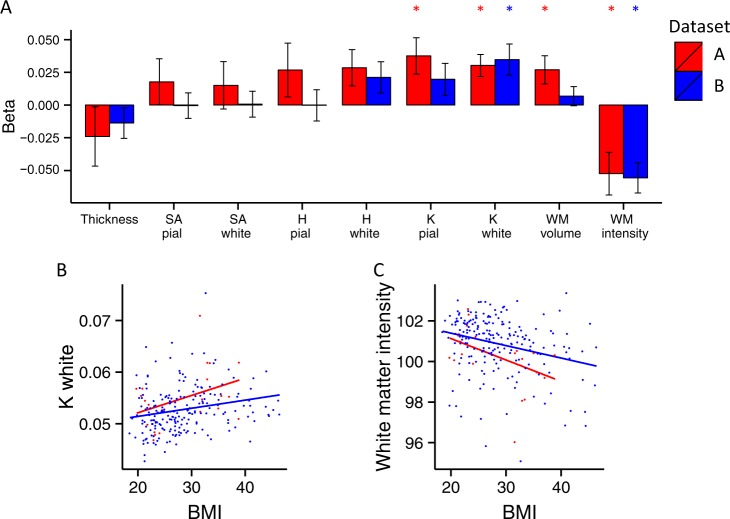
**a** Standardised regression coefficients from linear models exploring the association of subjects’ BMI (datasets A and B) with global morphometric measures of cortical thickness, surface area (SA) of the pial and white matter surfaces, mean curvature (H) of the pial and white matter surfaces, Gaussian curvature (K) of the pial and white matter surfaces, white matter (WM) volume and signal intensity. *p < 0.05 (FDR-corrected for nine tests). **b** Scatter plot of the association between Gaussian curvature at the white matter surface (K white) and BMI. **c** Scatter plot of the association between Gaussian curvature at the white matter signal intensity and BMI

There were no significant associations between BMI and measures of cortical thickness or surface area, either at the pial or white/gray matter interface in datasets A and B (Fig. 2, Supplementary table 1).

Mean curvature at the pial or white matter surface was not significantly related to BMI in either dataset. Gaussian curvature at the pial surface was positively associated with BMI in dataset A (β = 0.0376 ± 0.0139, *p* = 0.0093, *p*_FDR_ < 0.05), while no significant association with BMI was established in dataset B.

It was only the Gaussian curvature at the white matter surface that was consistently associated to BMI, in a positive direction, across both datasets (dataset A: β = 0.03 ± 0.084, *p* = 0.008, *p*_FDR_ < 0.05; dataset B: β = 0.0348 ± 0.0019, *p* = 0.0039, *p*_FDR_ < 0.05; Fig. 2b).

##### White matter

Global white matter volume was positively associated with BMI in dataset A (β = 0.027 ± 0.0108, *p* = 0.0155, *p*_FDR_ < 0.05), whereas no significant relationship between white matter volume and BMI was found in dataset B (Fig. 2, Supplementary table 1).

Across both groups of subjects, white matter signal intensity was negatively related to subjects’ BMI (dataset A: β = −0.0524 ± 0.0163, *p* = 0.0024, *p*_FDR_ < 0.05; dataset B: β = −0.0556 ± 0.0116, *p* *<* 0.0001, *p*_FDR_ < 0.05; Fig. 2c).

The influence of different scanner acquisition parameters in dataset B was investigated and indicated that these did not affect the presented results (see Supplementary information).

##### Regional analyses

To explore whether the association between global average Gaussian curvature at the white matter surface was driven by regional (i.e., lobar) effects, we computed average white matter Gaussian curvatures per each lobe, and examined their association with BMI. While there was some variability across lobes in their association with BMI, there was no significant BMI-by-lobe interaction in either of the two datasets (Table 1), suggesting that the global effect of BMI on white matter Gaussian curvature was not driven by regional effects.

**Table 1:** Standardised regression coefficients from linear models exploring the association of subjects’ BMI (datasets A and B) with Gaussian curvature at the white matter surface across cortical lobes

	Dataset A (*n* = 52)			Dataset B (*n* = 202)		
Lobe	Beta	SE	*p*	Beta	SE	*p*
Cingulate	0.0169	0.0085	0.0511	0.019	0.0051	0.0112
Fontal	0.034	0.0098	0.0012	0.0307	0.0114	0.0075
Insula	0.0353	0.0133	0.0106	0.0192	0.0085	0.0245
Occipital	0.0224	0.0091	0.0181	0.0331	0.0107	0.0023
Parietal	0.0299	0.0087	0.0012	0.0197	0.0109	0.0715
Temporal	0.0212	0.0085	0.019	0.0326	0.0097	0.0009

#### Mediation analyses

**Fig. 3:**
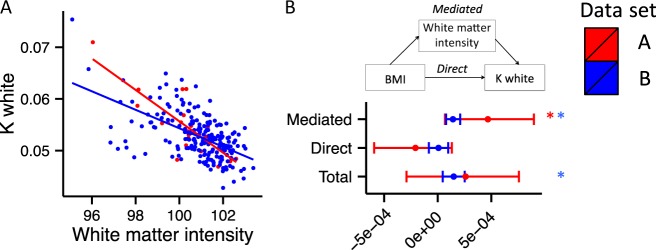
**a** Scatter plot of the association between white matter signal intensity and Gaussian curvature of the white matter surface (K white) (datasets A and B). **b** Graphical representation of the mediation analysis between BMI and K white, with white matter signal intensity as the mediator (datasets A and B): estimates of the mediated, direct and total effects. Error bars denote confidence intervals. *p < 0.05

The Gaussian curvature of the white matter surface was correlated with the intensity of white matter in both datasets (dataset A: *t* = −6.443, *p* < 0.0001; dataset B: *t* = −10.104, *p* *<* 0.0001; Fig. 3a). To test whether the effects of BMI on Gaussian curvature of the white matter surface were mediated by the effects of BMI on white matter microstructure, we conducted a mediation analysis. Across both datasets, the white matter signal intensity was established as a significant mediator of the association between BMI and Gaussian curvature of the white matter surface (ACME denotes the estimate of the mediated effect; dataset A: ACME = 0.00468, CI [0.0000681, 0.000897], *p* = 0.02; dataset B: ACME = 0.000142, CI [0.000078, 0.00021], *p* < 0.01; Fig. 3b).

### Dataset C

#### Regression analyses

**Fig. 4:**
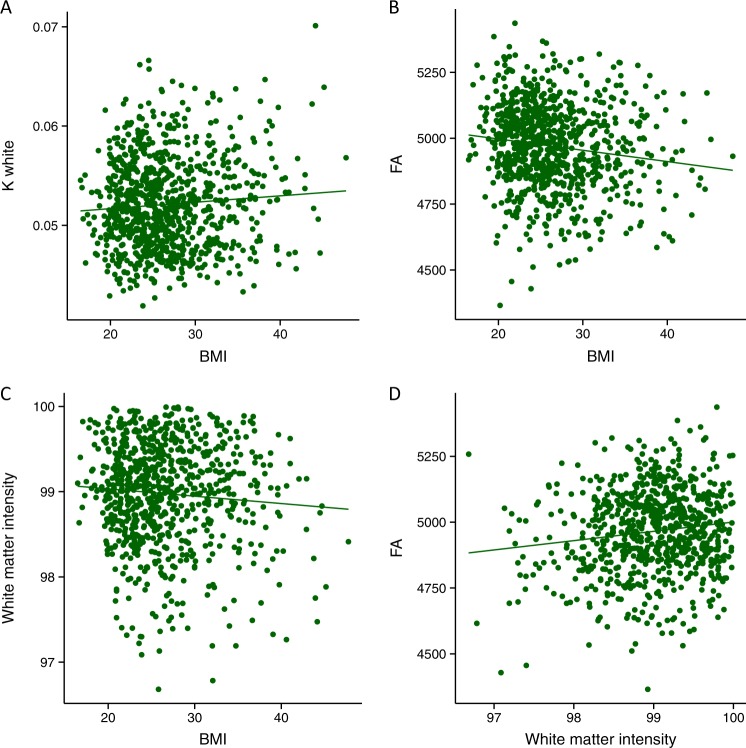
Scatter plots from dataset C, demonstrating the associations between: **a** BMI and Gaussian curvature of the white matter surface (K white); **b** BMI and FA; **c** BMI and white matter signal intensity; **d** white matter signal intensity and FA

Gaussian curvature of the white matter surface was positively associated with subjects’ BMI (β = 0.014 ± 0.064, *p* = 0.028, *p*_FDR_ < 0.05, Fig. 4a). White matter signal intensity demonstrated a trend-like negative association with BMI (β = −0.0119 ± 0.0061, *p* = 0.0536, Fig. 4b). For completeness, we conducted exploratory analyses examining the associations between BMI and all morphometric measures examined in datasets A and B (Supplementary table 3). Out of all the morphometric measures considered, it was only the association of BMI with Gaussian curvature at white matter surface that reached the threshold of *p* < 0.05. FA was negatively associated with BMI (β = −0.0206 ± 0.0058, *p* = 0.004, *p*_FDR_ < 0.05, Fig. 4c). FA was correlated with white matter signal intensity (*t* = 6.52, *p* < 0.0001, Fig. 4d).

#### Mediation analysis

**Fig. 5:**
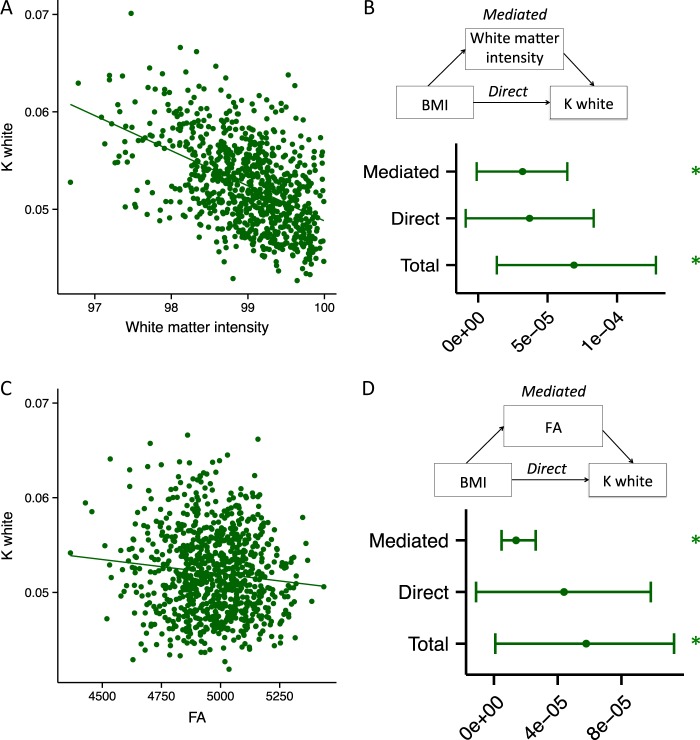
Mediation analyses in dataset C. **a** Scatter plot of the association between white matter signal intensity and Gaussian curvature of the white matter surface (K white). **b** Graphical representation of the mediation analysis between BMI and K white, with white matter intensity as the mediator: estimates of the mediated, direct and total effects. Error bars denote confidence intervals. **c** A scatter plot of the association between FA and Gaussian curvature of the white matter surface (K white). **d** Graphical representation of the mediation analysis between BMI and K white, with FA as the mediator: estimates of the mediated, direct and total effects. Error bars denote confidence intervals. *p < 0.05

White matter signal intensity was negatively correlated with Gaussian curvature of the white matter surface (*t* = −19.4892, *p* < 0.0001, Fig. 5a), and was a significant mediator of the association between BMI and Gaussian curvature of the white matter surface (ACME = 0.000032, CI [−0.00000107, 0.0000642], *p* *=* 0.05, Fig. 5b). These findings were in line with similar results from the previous two datasets. Critically, FA was negatively associated with Gaussian curvature of the white matter surface (*t* = −4.2793, *p* < 0.0001, Fig. 5c), and was identified as a significant mediator of the relationship between BMI and Gaussian curvature of the white matter surface (ACME = 0.00014, CI [0.0000048, 0.0000262], *p* = 0.01, Fig. 5d).

## Discussion

In this study we demonstrated that obesity is associated with small-scale morphometric changes in cerebral cortex and that differences in white matter integrity act as a mediator between obesity and cortical changes in three independent datasets of healthy young to middle-aged adults. While there was no consistent evidence of associations between BMI and global measures of cortical thickness, surface area or white matter volume, in all three datasets we found a consistent positive association of BMI with Gaussian curvature of the white matter surface. This was found across the entire brain and we found no evidence of region-specific effects. In keeping with our model, a mediation analysis demonstrated that the positive association between BMI and white matter surface Gaussian curvature was in turn associated with BMI-associated impairments of white matter microstructure.

Associations between brain structure and BMI have been inconsistent in the literature and this may relate to the methodologies as well as the scale of the measurement [3, 10]. We find that at smaller scales, there are clear associations between brain structure and BMI and demonstrate this across three independent datasets. Our findings therefore demonstrate the value of applying morphometric measures such as Gaussian curvature as an easily accessible marker of BMI-linked structural changes in future T1-weighted MRI studies. Though we demonstrate that this BMI-related increase in Gaussian curvature is mediated by white matter, the white matter signal intensity, as a proxy measure for white matter integrity obtainable from T1-weighted MRI, was not an equally reliable indicator of BMI-linked brain structure changes, given that in dataset C it only showed a trend-like association with BMI. Therefore, based on previous reports [10] and our findings, it can be concluded that in addition to FA in diffusion imaging, Gaussian curvature constitutes a usefully sensitive morphometric indicator of BMI-linked changes in brain structure and can be obtained from standard T1-weighted MRI scans.

In contrast to the measure of mean curvature, which describes the degree to which the cortical surface is folded, Gaussian curvature provides a measure of the intrinsic shape of the surface [11, 12]. Strikingly, it showed a consistent BMI-associated global alteration across 3 independent datasets, acquired on different scanners, with different scanning acquisitions as well as differences in number of individuals per dataset and exclusion criteria. This consistency illustrates the robustness of this parameter to BMI-related group differences. As discussed elsewhere [12, 18], Gaussian curvature may arise, either through non-uniform expansion or surface stretching/compression, and as such indexes the intrinsic form of the surface—i.e, the innate characteristics of the surface. By extension, Gaussian curvature provides a more fundamental and sensitive measure of critical aspects of cortical morphometry compared with parameters based on its extrinsic shape, i.e., folding. This point has previously been illustrated in relation to neurodevelopmental changes in schizophrenia [13]. In contrast to the Gaussian-curvature based results, the inconsistent and null results of other morphometric, volumetric and surface-based results highlights the lack of sensitivity of these parameters, and the increased potential to identify false positives. Ultimately these results underline the subtle nature of BMI-related differences in brain structure and the need to replicate findings across multiple datasets.

Having identified and internally replicated this relationship between BMI and curvature, the key question is what mechanical changes might account for it. Based on prior observations in neurodegenerative disorders [21], and the fact that our effects were observed on the white matter cortical surface rather than the pial surface, we hypothesised that this would be mediated by altered integrity of underlying white matter. We tested this hypothesis by relating white matter surface Gaussian curvature to two measures of white matter microstructure, namely the global average T1 white matter signal intensity (available in all three datasets), and global average FA (available in dataset C). This analysis demonstrated that the BMI-linked changes in the geometry of white matter surface were indeed mediated by the effects of BMI on white matter microstructure. The demonstration of cortical surface geometry as a function of the state of underlying white matter is consistent with previous studies of patient groups. Im et al [22]. reported that the lower mean curvature in sulci of subjects with mild cognitive impairment and Alzheimer’s disease was linked to reduced cortical thickness and white matter volume in gyri. Furthermore, in subjects with mild traumatic brain injury, increased global mean curvature was shown to be associated with increased white and gray matter volumes in the sulci, and reduced white and gray matter volumes in gyri [23]. Thus, there is a precedent for showing that small-scale changes in surface morphometry may reflect underlying white matter structural changes. The findings here, based on two characterisations of white matter integrity across three independent groups, provide strong evidence that this may also be the case in obesity. Our findings therefore are consistent with a model whereby the progression of adiposity-related brain changes starts with a reduction in white matter integrity. This phenomenon may in turn be subtly reflected in cortical morphometric change, albeit one that is only reliably quantified using small-scale measures and, even then, only found on the cortical surface most proximal to the white matter.

The association between white matter integrity and increased adiposity as reported here may be linked to various obesity-related pathophysiological mechanisms. Obesity-related inflammation is a one strong candidate, and it is well established that obesity is associated with low-grade systemic inflammation. For example, hypertrophic adipocytes have been linked to increased secretion of pro-inflammatory cytokines, such as IL-6 and TNF-alpha. These have been shown to induce an inflammatory response in the microglia with local cytokine release [24–27] and a resultant increase in white matter water content and reduction in white matter integrity [28, 29]. There are however other accompaniments of increased adiposity that can affect white matter integrity. Hypertension shares genetic risks with perturbed structure and function of the cerebral vasculature, increasing the risk of chronic hypoperfusion [30] and associated atrophic changes in white matter [27–30]. Hyperglycaemia may increase the production of reactive oxygen species, and has been implicated in detrimental vascular changes [31]. Further, dyslipidaemia and abnormal cholesterol profile have also been implicated as contributors to changes in white matter integrity [32]. However, these physiological factors are intricately linked to each other, and the extent to which they individually contribute to white matter has not been well established. For example, Sala et al [33]. listed triglycerides, BMI, and diastolic blood pressure as independent factors negatively contributing to white matter integrity. However, a separate study reported a negative association of markers of inflammation and hyperglycaemia with white matter integrity, but also a positive association of dyslipidaemia and blood pressure with white matter integrity [29]. These conflicting findings suggest a complex pattern of BMI-linked physiological influences on white matter integrity. This is unsurprising, not least given that these process are likely to proceed in parallel, albeit at different rates.

The MRI datasets analysed in this paper differed in demographic factors, and were collected at different research sites and by different research groups. Consequently, the inclusion and exclusion criteria were not consistent across datasets. While these differences between our three groups of subjects were advantageous in demonstrating the robustness of Gaussian curvature in detecting BMI-effects across different datasets, the availability of DTI only in dataset C restricted the type of analyses we could perform. However, across all three datasets, we extracted T1 white matter signal intensity at the distance of 1 mm below the white mater surface, as an indicator of white matter microstructure, and cross-validated this measure with global FA in dataset C, as a well-established measure of white matter integrity. The reduction of white matter signal intensity is related to increased T1 relaxation time, often driven by increased white matter water content [34], which is a non-specific indicator of a diseased state of white mater [35]. FA, on the other hand, is the most commonly used indicator of white matter integrity, derived from MRI-based inference of water diffusion within white matter. It is believed to indicate fibre density, axonal diameter or myelination of white matter [9]. Therefore, in the absence of other information, FA is a sensitive but a non-specific marker of white matter integrity. While we are careful in drawing conclusions about the correspondence of these two types of measures, we have demonstrated that FA and white matter signal intensity were correlated in dataset C, and that across all three datasets reduced white matter signal intensity mediated the effect between BMI and increased white matter Gaussian curvature, similarly as was the case with FA in dataset C. We acknowledge, however, that while the mediation analysis suggests that the association between BMI and Gaussian curvature is driven by effects on white matter microstructure, it cannot unequivocally establish the causality of this effect, which is an inherent limitation of a cross-sectional study. Further longitudinal-based work is required to assess the validity of this model. Finally, it must be acknowledged that results presented here were also acquired at two different magnetic field strengths. While the consistency of the results in the face of this variation in field is reassuring with respect to the reported effects, it is important to bear this in mind as a potential source of noise.

In summary, we have demonstrated a consistent increase in global Gaussian curvature at the white matter surface with increasing BMI in three datasets, including 1151 subjects in total. This effect was mediated by reduced white matter microstructure with increasing BMI. Our results suggest that BMI is associated with changes in white matter microstructure in young to middle-aged healthy adults, which is linked to changes in the intrinsic geometry of the white matter surface. We propose that Gaussian curvature can be used as a sensitive T1-weighted MRI measure for tracking the effects of obesity on cortical morphology.

## Electronic supplementary material

Supplementary infomation
