# Supplementary material for: BMI-related cortical morphometry changes are associated with altered white matter structure
Source: Int J Obes (Lond). 2018 Dec 19;43(3):523–32. doi: 10.1038/s41366-018-0269-9 (PMC6462878; doi:10.1038/s41366-018-0269-9)
Supplement: Supplementary file 1 — Supplementary infomation [file 41366_2018_269_MOESM1_ESM.pdf]

BMI-related cortical morphometry changes are associated with altered white matter structure

Medic et al.

Supplementary information

Supplementary methods

MRI processing and morphometric measures

Cortical reconstructions of T1-weighted scans (datasets A, B and C) were made in FreeSurfer

T1-weighted scans (datasets A, B and C)

Cortical reconstructions were made in FreeSurfer. For the MRI scans of first two groups of subjects, raw image data voxels were sub-sampled to isotropic resolution of 1 mm^3 using sinc interpolation. Radio-frequency bias inhomogenieties were modelled and removed, and skull-stripping was performed. The white matter surface was identified, hemispheres separated, tessellated and deformed, and a smooth and accurate representation of the pial and white matter surfaces was created. Reconstruction inaccuracies were edited manually. MRI scans from the third group of subjects were pre-processed using a modified version of the FreeSurfer pre-processing pipeline, as described in (1).

Measures of global average cortical thickness, total surface area (of white matter and pial surfaces) and total white matter volumes were extracted from FreeSurfer. Vertex-wise white matter intensities were extracted at 1mm distance from the white matter surface along surface normal towards white matter (2), and global average was computed. Mean and Gaussian curvature per each vertex of the cortex on the white matter and pial surface reconstructions were calculated in the software Caret (v5.65, http://brainmap.wustl.edu/caret), as the product and mean of principle curvatures, respectively. Global cortical average of absolute values of mean and Gaussian curvatures were then calculated in Matlab (3).

DTI scans (dataset C)

DTI data were pre-processed in the HCP Diffusion Pipeline (1). Briefly, b0 image intensity was normalised across runs, images were corrected for subject motion, EPI susceptibility and eddy currents, and a brain mask was applied. The diffusion model was fitted in the FSL-FDL toolkit to obtain the FA images. The ENIGMA-DTI protocol, described in (4) and available online (http://enigma.ini.usc.edu/ongoing/dti-working-group/), was used to extract the whole-brain average

FA values. This entailed using the FSS FNIRT to nonlinearly align the FA images to the ENIGMA-DTI minimum-deformation target brain.

The data were further processed using FSL's tract-based spatial statistics (https://fsl.fmrib.ox.ac.uk/fsl/fslwiki/TBSS), which was modified to project individual FA values on the hand-segmented ENIGMA-DTI skeleton mask. A global average was calculated across all voxels in the ENIGMA-DTI skeleton.

Supplementary results

Supplementary table 1. Outputs from linear models exploring the association of subjects' BMI (datasets A and B) with global morphometric measures of cortical thickness, surface area (SA) of the pial and white matter surfaces, mean curvature (H) of the pial and white matter surfaces, Gaussian curvature (K) of the pial and white matter surfaces, white matter (WM) volume and signal intensity. Standardised regression coefficient (beta), standard error (SE), degrees of freedom (df), coefficient of determination adjusted for the number of predictors (R^2 (adj.)); * $p < 0.05$ (FDR-corrected for nine tests).

	Dependent variable	Beta	SE	p	F (model)	df	p (model)	R^2 (adj.)
Dataset A	Thickness	-0.0240	0.0227	0.2960	1.423	3, 48	0.241	0.0321
	SA pial	0.0178	0.0177	0.3181	10.01	3, 48	0.00	0.4140
	SA white	0.0151	0.0181	0.4090	9.044	3, 48	0.0000	0.3868
	H pial	0.0268	0.0206	0.1992	3.913	4, 47	0.0049	0.2221
	H white	0.0281	0.0138	0.0479	19.65	4, 47	0.00	0.6464
	K pial	0.0376	0.0139	0.0093*	19.72	4, 47	0.00	0.6473
	K white	0.0300	0.0084	0.0008*	69.15	4, 47	0.00	0.8698
	WM Volume	0.0270	0.0108	0.0155*	38.2	4, 47	0.00	0.7848
	WM intensity	-0.0524	0.0163	0.0024*	13.7	3, 48	0.00	0.4991
Dataset B	Thickness	-0.0137	0.0117	0.2427	5.03	4, 197	0.00	0.0742
	SA pial	-0.0004	0.0097	0.9654	29.62	4, 197	0.00	0.3629
	SA white	0.0007	0.0099	0.9453	26.86	4, 197	0.00	0.3398
	H pial	-0.0002	0.0119	0.9872	2.78	5, 196	0.0189	0.0424
	H white	0.0212	0.0119	0.0772	2.749	5, 196	0.0200	0.0417
	K pial	0.0197	0.0122	0.1090	0.703	5, 196	0.6218	0.0074
	K white	0.0348	0.0119	0.0039*	2.848	5, 196	0.0166	0.0440
	WM volume	0.0068	0.0073	0.3502	73.35	5, 196	0.0000	0.6428
	WM intensity	-0.0556	0.0116	0.0000*	6.512	4, 197	0.00	0.0989

Sensitivity analysis in dataset B (influence of different scanner acquisition)

To further demonstrate that the acquisition at two different scanners in dataset B did not affect the established association between BMI and measurers of gray and white matter, we split dataset B into two subsets, based on scanner, and then separately repeated the analysis (including the same covariates, other than scanner). The results obtained in each scanner-defined subset of dataset B were comparable to the results in the combined dataset (Supplementary table 2).

Supplementary table 2. Outputs from linear models exploring the association of subjects' BMI with global morphometric measures in two subsets of dataset B that were acquired on different scanners. Standardised regression coefficient (beta), standard error (SE), degrees of freedom (df), coefficient of determination adjusted for the number of predictors (R^2 (adj.)).

	Dependent variable	Beta	SE	p	F (model)	df	p (model)	R^2 (adj.)
Scanner 1 (n = 65)	Thickness	-0.0165	0.0216	0.4500	0.4952	3, 61	0.6869	-0.0242
	Surface area pial	0.0137	0.0153	0.3720	21.3700	3, 61	0.0000	0.4884
	Surface area white	0.0136	0.0158	0.3920	18.7700	3, 61	0.0000	0.4545
	H pial	0.0214	0.0213	0.3170	1.3800	4, 60	0.2517	0.0232
	H white	0.0361	0.0212	0.0942	1.4210	4, 60	0.2380	0.0256
	K pial	0.0464	0.0210	0.0310	1.7900	4, 60	0.1427	0.0471
	K white	0.0474	0.0211	0.0287	1.6010	4, 60	0.1857	0.0362
	WM volume	-0.0055	0.0107	0.6090	49.0600	4, 60	0.0000	0.7502
	WM intensity	-0.0470	0.0193	0.0176	5.9610	3,61	0.0012	0.1887
Scanner 2 (n = 137)	Thickness	-0.0104	0.0141	0.4618	6.5260	3,133	0.0004	0.1087
	Surface area pial	-0.0079	0.0126	0.5305	20.0100	3,133	0.0000	0.2954
	Surface area white	-0.0061	0.0128	0.6360	17.7700	3,133	0.0000	0.2700
	H pial	-0.0093	0.0150	0.5360	0.8497	4,132	0.4962	-0.0044
	H white	0.0150	0.0149	0.3170	1.5430	4,132	0.1935	0.0157
	K pial	0.0070	0.0152	0.6430	0.2964	4,132	0.8799	-0.0211
	K white	0.0274	0.0146	0.0627	3.0120	4,132	0.0205	0.0559
	WM volume	0.0120	0.0098	0.2210	46.7300	4,132	0.0000	0.5736

WM intensity	-0.0547	0.0144	0.0002	5.0620	3,133	0.0024	0.0822

Supplementary table 3. Outputs from linear mixed effects models exploring the association of subjects' BMI (dataset C) with global morphometric measures of cortical thickness, surface area (SA) of the pial and white matter surfaces, mean curvature (H) of the pial and white matter surfaces, Gaussian curvature (K) of the pial and white matter surfaces, white matter (WM) volume and signal intensity. Standardised regression coefficient (beta), standard error (SE), degrees of freedom (df), Akaike information criterion (AIC), Bayesian information criterion (BIC), log-likelihood (logLik).

	**Dependent variable**	**Beta**	**SE**	**df**	**p**	**AIC**	**BIC**	**logLik**	**R^2 (adj.)**
Dataset C	Thickness	0.0114	0.0060	512	0.0573	2403.477	2432.238	-1195.739	0.4757
	SA pial	-0.0011	0.0044	512	0.8038	1901.06	1929.821	-944.5299	0.7443
	SA white	-0.0039	0.0045	512	0.3866	1921.383	1950.144	-954.6916	0.7313
	H pial	-0.0089	0.0060	511	0.1373	2413.954	2447.501	-1199.977	0.4739
	H white	-0.0039	0.0057	511	0.4948	2329.235	2362.782	-1157.618	0.5736
	K pial	0.0006	0.0063	511	0.9269	2477.182	2510.729	-1231.591	0.3815
	K white	0.0141	0.0063	511	0.0273	2502.352	2535.899	-1244.176	0.3273
	WM volume	-0.0038	0.0033	511	0.2484	1347.108	1380.654	-666.554	0.8383
	WM intensity	-0.0118	0.0061	512	0.0547	2441.118	2469.879	-1214.559	0.4102
	FA	-0.0206	0.0058	481	0.0004	2224.579	2253.093	-1106.289	0.5426

References

1. Glasser MF, Sotiropoulos SN, Wilson JA, Coalson TS, Fischl B, Andersson JL, et al. The Minimal Preprocessing Pipelines for the Human Connectome Project. NeuroImage. 2013 Oct 15;80:105–24.

2. Salat D, Lee S, van der Kouwe A, Greve D, Fischl B, Rosas H. Age-Associated Alterations in Cortical Gray and White Matter Signal Intensity and Gray to White Matter Contrast. NeuroImage. 2009 Oct 15;48(1):21–8.

3. Ronan L, Voets N, Rua C, Alexander-Bloch A, Hough M, Mackay C, et al. Differential tangential expansion as a mechanism for cortical gyrification. Cereb Cortex N Y N 1991. 2014 Aug;24(8):2219–28.

4. Jahanshad N, Kochunov P, Sprooten E, Mandl RC, Nichols TE, Almassy L, et al. Multi-site genetic analysis of diffusion images and voxelwise heritability analysis: A pilot project of the ENIGMA–DTI working group. NeuroImage. 2013 Nov 1;81:455–69.
